# Hole-phonon coupling effect on the band dispersion of organic molecular semiconductors

**DOI:** 10.1038/s41467-017-00241-z

**Published:** 2017-08-02

**Authors:** F. Bussolotti, J. Yang, T. Yamaguchi, K. Yonezawa, K. Sato, M. Matsunami, K. Tanaka, Y. Nakayama, H. Ishii, N. Ueno, S. Kera

**Affiliations:** 1 Institute for Molecular Science, Myodaiji, Okazaki 444-8585 Japan; 2 Graduate School of Advanced Integration Science, Chiba University, Chiba, 263-8522 Japan; 3 SOKENDAI (The Graduate University for Advanced Studies), Hayama, Kanagawa 240-0193 Japan; 4 Institute of Materials Research and Engineering (IMRE), 2 Fusionopolis Way, Innovis, Singapore, #08-03 Singapore; 5 College of Physical Science and Technology, Yangzhou University, Jiangsu, 225009 People’s Republic of China; 6 Toyota Technological Institute, 2-12-1 Hisakata, Tempaku-ku, Nagoya 468-8511 Japan; 7 Department of Pure and Applied Chemistry, Faculty of Science and Technology, Tokyo University of Science, 2641 Yamazaki, Noda-shi, Chiba-ken 278-8510 Japan

## Abstract

The dynamic interaction between the traveling charges and the molecular vibrations is critical for the charge transport in organic semiconductors. However, a direct evidence of the expected impact of the charge-phonon coupling on the band dispersion of organic semiconductors is yet to be provided. Here, we report on the electronic properties of rubrene single crystal as investigated by angle resolved ultraviolet photoelectron spectroscopy. A gap opening and kink-like features in the rubrene electronic band dispersion are observed. In particular, the latter results in a large enhancement of the hole effective mass (> 1.4), well above the limit of the theoretical estimations. The results are consistent with the expected modifications of the band structures in organic semiconductors as introduced by hole-phonon coupling effects and represent an important experimental step toward the understanding of the charge localization phenomena in organic materials.

## Abstract (web summary)

The charge transport properties in organic semiconductors are affected by the impact of molecular vibrations, yet it has been challenging to quantify them to date. Here, Bussolotti et al. provide direct experimental evidence on the band dispersion modified by molecular vibrations in a rubrene single crystal.

## Introduction

In recent years, the use of organic molecular semiconductors as base materials for optoelectronic applications marked a significant advancement in the technological field, resulting in an entirely new class of light, flexible and low-cost devices^1–3^. Despite the technological achievements, important aspects of the physics of these materials remain still elusive, as for example the exact nature of the charge transport mechanism. This important issue was originally addressed by applying concepts and ideas developed in transport studies of inorganic semiconductors and metals. In this context, the charge transport in organic molecular semiconductors was described with respect to the limiting case of coherent band-like transport and incoherent hopping-like transport^4^ corresponding to the extreme delocalization and localization of the charge carriers, respectively. The former mechanism is dominated by the energy dispersion of the highest occupied (HOMO) and lowest unoccupied (LUMO) molecular orbital derived bands which reflects the spatial overlap of the molecular wavefunctions^4^. The latter mechanism is affected by the charge reorganization energy which depends on the hole/electron coupling with the intramolecular vibrations, as molecules generally undergoes structural relaxations upon receiving additional charges^4, 5^. However, both the conventional band and hopping transport theories soon appeared inadequate for describing the measured charge mobility in a large number of organic materials^6^. As a matter of fact, the charge localization/delocalization phenomena in molecular organic semiconductors occur on comparable characteristic energy scale (~50–200 meV^4^) which prevents the applicability of the above mentioned conventional limiting treatments^6^. More realistically, the charge transport mechanism in molecular organic semiconductors is expected to result from the delicate interplay between charge spatial delocalization vs. localization processes^6–8^ both affected, in turn, by the details of the crystal structure and temperature^4, 6–8^. Intense theoretical research is still on-going to include all these critical aspects in a fully consistent and accurate description of the charge carrier dynamics in organic molecular semiconductors^6–8^. More recently, the hole/electron coupling with intermolecular vibrations was also discussed as a possible origin of charge localization, as related to the continuous modulation of the spatial overlap between molecular wavefunctions^7–10^.

In this context, experimental studies on the impact of the charge-phonon coupling on the electronic properties of the organic molecular semiconductors may provide critical information (i.e., coupling strength, phonon energies, and so on) for refining the details of the various transport models and/or testing the validity of the corresponding predictions.

In metals, the charge coupling with lattice phonons manifests in local change in the curvature of the band dispersion (i.e., kink structures), as directly revealed by a number of angle resolved ultraviolet photoemission spectroscopy (ARUPS) investigations^9^. The kink position and the degree of the related band distortion reflect the energy of the phonons and the strength of their coupling with the charge carriers. These effects may result, for example, in the enhancement of the carrier effective mass at the Fermi level, with consequent impact on the charge mobility^9^. Local change in the band curvature was similarly predicted to appear in the band dispersion of organic semiconductors, originating from the charge coupling with molecular vibrations^11^. However, despite theoretical predictions and relevance for applications, a direct experimental evidence of band distortions in organic molecular semiconductors as mediated by the charge-phonon coupling has yet to be observed.

In this paper, we report on the ARUPS investigation of the band dispersion of rubrene single crystal. With one of the highest reported hole mobility value (~40 cm^2^/Vs)^12^ among molecular organic semiconductors, rubrene single crystal represents a prototype system for both fundamental and applicative studies^6–8, 12–15^. A gap opening and kink-like distortion of the HOMO derived band dispersion are revealed. The results are consistent with the expected modifications of the band structures in organic semiconductor as introduced by hole-phonon coupling effects, as predicted by theoretical calculations on organic models system^11^. This result directly confirms the leading role of molecular vibrations in affecting the charge localization in organic single crystals, which is critical for charge mobility^6–8, 12–15^.

## Results

### ARUPS measurements of the HOMO band dispersion

**Fig. 1:**
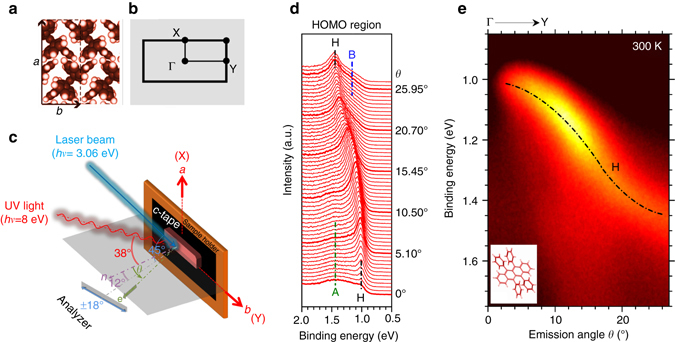
Crystal structure with ARUPS experimental geometry and data of rubrene single crystal. Schematic of the molecular orientation in the crystalline *a*-*b* plane of rubrene single crystal (*a* = 14.4 Å, *b* = 7.2 Å) in real **a** and reciprocal space **b**. **c** Schematic drawing of the experimental geometry. **d** *θ* dependence of the ARUPS spectra of rubrene at 300 K along the ΓY direction. The peak positions of the HOMO (H) and of non-dispersive features **a** and **b** are indicated by *dash dotted vertical lines* (see text for further details). **e** ARUPS spectra intensity as a function of the emission angle *θ* along the ΓY direction. The HOMO (H) dispersions is indicated by dash dotted black curve as guides for the eye. A sketch of the rubrene molecule is reported in the inset for reference

The rubrene crystal structure in real and reciprocal space are described in Fig. 1a, b, respectively, while the ARUPS experimental geometry is schematically depicted in Fig. 1c.

Figure 1d shows the *θ* dependence of the ARUPS spectra in the HOMO binding energy region of a rubrene single crystal, as acquired at 300 K along the ΓY high symmetry direction (Fig. 1b). In real space, the ΓY direction corresponds to the *b* axis (Fig. 1a) of the rubrene unit cell along which the highest hole mobility values were reported^12, 14, 15^. The corresponding ARUPS intensity map is shown in Fig. 1e. In the ARUPS spectra a peak like, energy dispersive feature (H) is clearly visible [Fig. 1d, e]. At low emission angle (*θ* ≤ 5°) the intensity of the H peak rapidly decreases near the Γ point (*θ* = 0°). The attenuation can be qualitatively explained in terms of photoemission matrix effect, reflecting the UV light polarization and the symmetry of the initial (HOMO) and final (photoelectron) states as coupled by the dipole transition^16^. This effect was previously reported to modulate the intensity of the ARUPS spectra of crystalline materials around the high symmetry points of the reciprocal space^17^. Close to the Γ point a broad, non-dispersive spectral feature (A) is also observed, as centered at ~1.4 eV of binding energy. At higher emission angle (*θ* > 20°) a shoulder (B) appears at ~1.25 eV, located on the low binding energy tail of the HOMO peak H.

### Rubrene HOMO band dispersion at 300 K

**Fig. 2:**
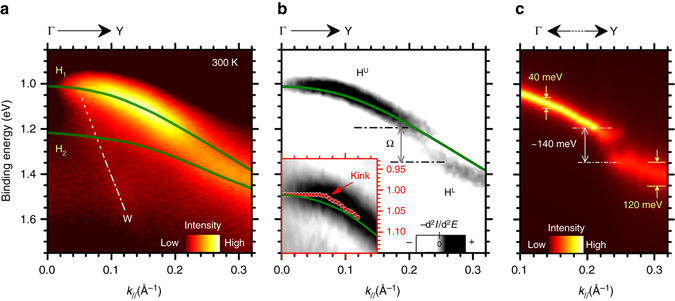
Angle resolved band map at 300 K and theoretical calculations. **a** ARUPS intensities vs. *k*_*//*_ along the ΓY direction of rubrene single crystal at 300 K. Theoretical HOMO band dispersions are plotted as continuous *green lines*. **b** Second derivative ARUPS intensity map (–d^*2*^I/d^*2*^ *E*, where *I* is the ARPES intensity and *E* the binding energy) as obtained from data in **a**. The splitting of the HOMO band in two subband H^U^ and H^L^ is evidenced as separated by gap Ω. Inset: Magnification of second derivative map close to Γ point. Experimental H peak positions are also indicated by *red circles* and compared with theoretical band dispersion (continuous *green line*) to highlight the kink-like distortion. **c** Intensity map of H peak Lorentzian component along the ΓY direction, as extracted from HOMO peak fitting procedure. The intensities of Lorentzian components were normalized with respect to the peak area

The HOMO-ARUPS intensity map as a function of the in-plane wave vector component (*k*_//_) along ΓΥ is reported in Fig. 2a. The calculated HOMO bands [as extracted from ref. ^18^] are overlaid as continuous lines to the experimental data. Two HOMO derived bands (H_1_ and H_2_) were theoretically predicted^18^. The HOMO band splitting originates by the presence of two structurally inequivalent molecules in the in-plane rubrene unit cell (Fig. 1a)^18^ as similarly reported for other organic molecular semiconductors^4^.

The calculated H_1_ band reproduces quite well the H band dispersion, while no clear experimental evidence of H_2_ band is found. Moreover, the presence of A and B feature is not supported by the theoretical calculations. These differences are discussed in Supplementary Note 1 as side aspects of the hole-vibration coupling mechanism demonstrated in the present work (Supplementary Fig. 1a, b).

Finally, a rapidly dispersive *w* band is observed, which was assigned to photoelectron scattering into unoccupied states of rubrene single crystal. A detailed discussion on these effects is included in Supplementary Note 2 with the support of the experimental data reported in Supplementary Figs 2–4.

While the overall behavior of H band dispersion is quite consistent with the theoretical calculations, small but finite differences exist as revealed by the second derivative analysis of the ARUPS data (Fig. 2b and inset). In particular, a gap-like structure is observed at ~0.22 Å^−1^ with the H band splitted into an “upper” (H^U^) and “lower” (H^L^) subband (Fig. 2b) and a kink-like feature is observed in the experimental band dispersion near the Γ point (inset of Fig. 2b).

The HOMO energy distribution curves (EDCs) at different *k*_*//*_ values were analyzed by least square peak fitting procedure, with the H peak simulated by a Voigt function according to the procedure described in Methods section. The as-extracted Lorentzian components of H peak, which reflects the hole quasiparticle dynamics^16^, were reported at variance of *k*_*//*_ in the color intensity map of Fig. 2c (EDC fitting analysis in Methods section). A value of Ω = 140 ± 20 meV was estimated from the H^U^ and H^L^ peak separation in the [0.20 ~ 0.22 Å^−1^] wave vector range. Upon crossing the energy gap, the Lorentzian full width at half maximum (*w*_L_) increases from 40 meV (H^U^ band) to 120 meV (H^L^ band) (Fig. 2c) which corresponds to a decrease of the hole lifetime *τ* ( = *ħ/w*_L_^14^).

The data reported in Fig. 2b may suggest a possible identification of the H^U^ and H^L^ subbands with H_1_ and H_2_ theoretical band, respectively, their intensity being suddenly modulated by photoemission matrix element effect. This possibility is excluded on the basis of the theoretical and experimental arguments presented in Supplementary Note 1.

### Rubrene HOMO band dispersion at 110 K

**Fig. 3:**
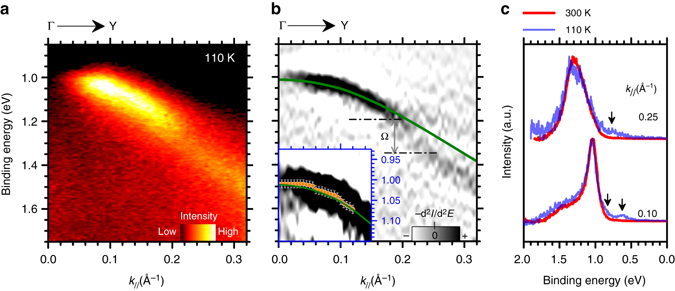
Angle resolved band map at 110 K and theoretical calculations. **a** ARUPS intensities vs. *k*_*//*_ along the ΓY direction of rubrene single crystal at 110 K; **b** Second derivative ARUPS intensity map (–d^*2*^I/d^*2*^ *E*, where *I* is the ARPES intensity and *E* the binding energy) as obtained from data in **a**. The splitting of the HOMO band in two subband H^U^ and H^L^ is evidenced as separated by gap Ω. Inset: Magnification of second derivative map close to Γ point. Experimental H peak positions are also indicated by *orange circles* and compared with theoretical band dispersion (continuous *green line*) **c** HOMO-EDC curves (Shirley background subtracted, intensity normalized at the curve maximum) at *k*_*/*/_ = 0.10 Å^−1^ and *k*_*/*/_ = 0.250 Å^−1^, as obtained at 300 K (*red line*) and 110 K (*blue line*). Peak like/tailing structure (*arrow*) are related to structural defects resulting from the temperature-induced mechanical stress in the rubrene single crystal

ARUPS measurements were repeated upon cooling rubrene single crystal to 110 K (Methods section). The corresponding ARUPS intensity map and the results of second derivative analysis are plotted in Fig. 3a, b, respectively. At low temperature the kink-like feature near Γ point is no longer visible, with the curvature of the H band well reproduced by the theoretical calculations. With respect to 300 K measurements, no significant changes in the energy gap size and position were detected, as well in the energy width of the HOMO-EDCs (Fig. 3c). A comparison between the EDCs as obtained at 300 and 110 K in the [0 ~ 0.095]Å^−1^ wave vector range is shown in Supplementary Fig. 5 to illustrate the temperature-induced binding energy shift resulting in the quenching of the kink structure.

### Gap opening in the HOMO band dispersion

The impact of the molecular vibrations on the electronic properties of organic materials was experimentally investigated by ARUPS for thin films of weakly interacting molecules^19^ where non-dispersive HOMO bands consisting of several components with comparable energy separations were observed^19^. Under simplified assumptions (single mode analysis), this energy separation (Ω_0_, typically ≳ 100 meV) corresponds to the average intramolecular vibration (local hole-phonon coupling effect) energy while the relative intensities of the HOMO components reflects the coupling strength^19^. This approach was theoretically generalized to the case of molecular crystals where larger HOMO bandwidths (*W*) are expected^11^. When *W*≳Ω_0_, the vibrational structure characteristic of the thin film photoemission spectra is replaced by “cuts” in the energy band dispersion. Spectral intensity modulation is thus introduced along the energy axis and gap-like structures appears across the HOMO band dispersion^11^. The energy gap size and position in the energy/momentum space reflect the value of the intramolecular vibration energies and the strength of the corresponding hole-phonon coupling, as discussed in the theorethical background of Supplementary Note 3. In this context, the gap-like feature in H band dispersion (bandwidth ~ 0.52 eV) of rubrene single crystal (Fig. 2a, b) can be related to the hole coupling with intramolecular vibrations with an average energy of ~140 meV. An average intramolecular vibrational energy of ~130 meV was extracted by the single mode analysis of ARUPS-HOMO line shape for rubrene thin film, in good consistence with the present result^20^.

The local nature of the hole-phonon coupling process explains the rapid change in the H peak width (and related hole lifetime) upon gap crossing along the ΓY direction (Fig. 2c). When a hole is created in the H^U^ band, the electrons at lower binding energies cannot be scattered into the empty electronic states by intramolecular phonon activation process, as the energy difference between the initial and final electron states is lower than the intramolecular vibration energy (~140 meV). The suppression of the scattering mechanism results in a large hole lifetime. The situation is reversed in the lower H^L^ band where the electron scattering as mediated by intramolecular phonon activation is energetically possible, thus leading to decreasing of the hole lifetime.

Finally, as shown in Fig. 3 the gap size and H peak width are not affected by the sample temperature. In (110–300 K) range, in fact, the thermal energy *kT* (< 25 meV) is much lower with respect to the rubrene intramolecular vibrational energy (~140 meV). Therefore no intramolecular vibrations can be thermally excited/quenched and their impact of the band dispersion and hole lifetime is thus largely temperature independent as confirmed by the experimental data.

### Kink structure in HOMO band dispersion

Kink-like structures were experimentally observed in the ARUPS measurements of metal conduction band and related to the hole coupling with the lattice vibrations^9^. The position of the kink with respect to Fermi level (i.e., the highest occupied level for metal) corresponds to the energy of the coupled vibrational mode^9^.

In analogy with the inorganic case, the kink structure near the top of the rubrene HOMO band (< 20 meV) at 300 K (inset Fig. 2b) can be related to the hole coupling with low energy intermolecular vibrations (non-local hole-phonon coupling) of rubrene single crystal.

**Fig. 4:**
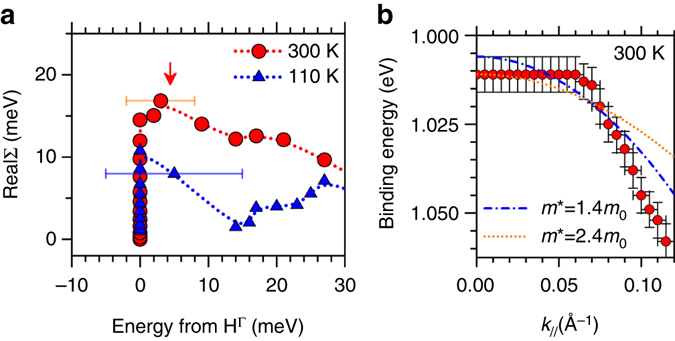
Real part of Self Energy and hole effective mass. **a** Real part of quasiparticle self-energy (Σ) at 300 and 110 K (see inset in Figs 2b and 3b). The energy values were evaluated with respect to the H peak position at Γ point (H^Γ^), the error bar being ±5 meV (at 300 K) and ±10 meV (at 110 K) as resulting from the EDCs peak fitting procedure **b** Experimental H band dispersion near Γ point region at 300 K, with corresponding energy error bars (±5 meV). The band curvature close to Γ point was estimated by means of parabolic curves centered at Γ within the [0–0.07] Å^−1^ wave vector range, where kink is observed, and corresponding effective mass value was extracted

From a careful comparison between the experimental data and theoretical calculations near the Γ point (see Methods section) the real part (RealΣ) of the hole quasiparticle self-energy (Σ) was extracted. The results of the analysis are reported in Fig. 4a. At 300 K, a peak at ~8 meV from the top of the HOMO band (HΓ) is visible. According to the general theory of photoemission spectral function^9, 16^ the position of the peak in the real part (RealΣ) of the quasiparticle spectral function corresponds to the energy of the (intermolecular) phonon involved in the hole-vibration coupling^9, 16^. Strongly coupled intermolecular vibrational modes were predicted at 4–16 meV by phonon spectra calculation of rubrene single crystal^8^, in good agreement with the present findings.

At 110 K no peak like feature is observed in the extracted RealΣ (Fig. 4a), which corresponds to the disappearance of the kink-like structure in the low temperature ARUPS data (inset of Fig. 3b). The low energy intermolecular vibrations are expected to be rapidly quenched by the temperature reduction as their energies is comparable with *kT* value in [110–300 K] temperature range ( < 25 meV). In this context, the temperature dependent ARUPS study supports the local nature of the hole-coupling effect near the top of the rubrene HOMO band.

## Discussion

Our ARUPS investigation can help to clarify important aspects of the hole dynamics in rubrene single crystal, which are crucial for the charge transport mechanism. As previously discussed, the hole lifetime in the upper H^U^ band (originating from local hole-phonon coupling effect) is not affected by the scattering with intramolecular phonons (Fig. 2c). Since only the hole states near the HOMO band edges can be thermally populated or doped in any real organic-based device^15^, it is possible to conclude that the scattering from high energy intramolecular modes does not play a significant role in the observed charge transport properties of rubrene single crystal.

Near the top of the HOMO band, the non-local hole-phonon coupling leads to a change (i.e., decrease) of the band curvature d^2^ *E(k*_*//*_ *)*/d^2^ *k*_*//*_ with respect to the results of the theoretical calculation. This directly affects the value of the hole effective mass *m* ^*^ (as *m* ^*^ ∝ 1/(d^2^ *E(k*_*//*_ *)*/d^2^ *k*_*//*_)^4^ which is an essential parameter to define the charge mobility (*μ*) in both inorganic and organic systems^4, 9^. The band curvature was measured by parabolic fitting of the experimental HOMO dispersion near the Γ point, as shown is Fig. 4b (see Methods section). A value of m* > 1.4 *m*_0_ was obtained (where *m*_0_ is the hole mass at rest) which is well above the theoretical value (0.8 ~ 0.9 *m*_0_) as estimated on the basis of band structure calculations and commonly used to in charge transport model of rubrene single crystal^10, 15, 18^.

Despite the conceptual limit of the band model discussed in the introduction, the increase of the hole effective mass (m* > 1.4 *m*_*0*_) rationalizes quite well the theoretical overestimation of the rubrene hole mobility with respect to the experimental value, as obtained by band-like transport model where *μ* ∝ 1 /*m* ^*^ with m* = 0.8 ~ 0.9*m*_0_^10, 15, 18^. The effective mass enhancement quantitatively describes the hole localization process as related to the charge coupling with intramolecular vibrations of rubrene lattice^8^.

The hole localization effects in rubrene single crystal are basically quenched as 110 K, where the band dispersion near the HOMO edge positions are well described by theoretical calculations (Fig. 3b). This result is consistent to the recovery of band transport limit and the increase of the intrinsic hole mobility at low temperature, as reported for rubrene and other organic crystalline system^13^.

In the present research, we report a rigorous experimental investigation on the electronic properties of rubrene single crystal, a prototypical example of organic semiconductor. We provided a clear evidence of peculiar features in the band dispersion which are not predicted by usual theoretical band structure calculations. In analogy with ARUPS theory, this deviation strongly resembles the effect of charge-phonon coupling in one-hole state as commonly reported in ARUPS investigation of inorganic single crystal. The temperature dependent data supported this interpretation. Moreover this is also consistent with the current state of the art of hole quasiparticle spectral function calculation in organic systems, with obvious deviations as related to the difference between our investigated system and the simplified assumption in the theoretical models. We also provide direct evidence on how charge-phonon coupling may increase charge localization, well above the limit predicted by DFT band structure calculation, which is a relevant result for applications. A complete simulation of the quasiparticle spectral function with theoretical calculation of the UPS spectra as well as a more quantitative modeling of charge transport in organic materials will help in a full rationalization of the present experimental results. In this context, the present work can attract interest of theoretical groups and stimulate the theoretical debate thus representing an important step towards the full understanding of the impacts of charge-phonon coupling on the electronic and transport properties of organic semiconductor crystals.

## Methods

### Synthesis of single crystal and sample preparation

Rubrene single crystals were prepared by physical vapor transport technique under nitrogen stream. Elongated sample of similar lateral size (~2 × 5 mm) and thickness (~200 μm) were mounted on a conductive carbon tape (STR-9181, Shinto Chemitron Co., LTD) positioned on a copper sample holder. Then, the edges of the single crystals were carefully covered with conductive carbon paste in order to improve the mechanical and electric contact with the underlying carbon tape.

### ARUPS measurements

ARUPS study was conducted at beamline BL7U of UVSOR synchrotron facility (Okazaki, Japan) according to the experimental geometry described in Fig. 1c. For all the investigated samples, comparable photoemission results were found. During the photoemission the sample were concurrently irradiated with continuous laser light (energy = 3.06 eV, power 10 mW) to overcome the sample charging effects induced by the ionizing UV radiation due to the high electrical resistance of organic single crystals. Under the present experimental conditions the sample charging was completely quenched as evaluated by preliminary ARUPS investigations and in agreement with previous experimental reports^21^.

In some of the prepared rubrene single crystals, the sample charging induced by the UV light irradiation was found to be considerably reduced, probably due to a better crystal quality and lower sample thickness. In these cases, a fast ARUPS data acquisition was also possible without concomitant laser light irradiation. These data were compared with ARUPS data obtained under laser light irradiation (not shown). The comparison indicates the absence of any significant effect of the laser light irradiation on the key features (i.e., kink, bandwidth, and so on) of the measured band dispersion of rubrene single crystal.

The UV light spot size on the sample was ~1 mm × 100 μm. Electron emission angle *θ* was measured with respect to the normal to the sample surface and photoelectrons were collected in the (−6°;+30°) emission angular range (Fig. 1c) at the angular resolution of ~0.16°. The binding energy value was referenced to the Fermi Level (*E*_F_) measured on a polycrystalline gold sample. The energy resolution was better than 10 meV as judged from the Fermi edge of the reference gold sample. The rubrene single crystals were oriented according to the anisotropy of crystal shape which reflects the symmetry of the crystal unit cell in real and reciprocal space^21^. All samples were measured as-inserted i.e., no sample cleaving was performed after its introduction into UHV environment. In the investigated photoelectron kinetic energy range (*E*_kin_ < 8 eV) the estimated electron mean free path is ~3 nm^22^ which corresponds to several rubrene layers. The ARUPS data therefore largely reflect the electronic properties of the bulk region and are rather insensitive to the structural and chemical defects of the surface region due to gaseous contamination and/or photo oxidation process^23^.

For temperature dependent ARUPS study the sample was slowly cooled (~18 h) from 300 to 110 K. The cooling rate was kept slow in order to minimize temperature-induced mechanical stress in the crystal and at the contact between sample and conductive paste/tape. Temperature-induced defects in the single crystal resulted in localized states in the rubrene energy gap as shown by the peak like and tailing feature in the low temperature HOMO lineshape^21^.

During the low temperature ARUPS measurement, the incident photon flux was ~4 times reduced to compensate the decrease of the electrical conductivity of carbon paste and tape at low temperature and avoid sample charging.

### EDCs fitting analysis

The HOMO-EDCs at various *k*_*//*_ was analyzed by least square fitting procedure. The H peak was simulated by Voigt function. The FWHM of the Gaussian component *w*_G_ was set within [130 ~ 150] meV to reproduce the Gaussian broadening which originates from (i) relaxation/polarization effect resulting from structural disorder and (ii) experimental resolution^19^. The A and B features was simulated by superimposition of Gaussian peaks. Representative fitting results of EDCs at various *k*_*//*_ are reported in Supplementary Fig. 6.

In obtaining the plot of Fig. 2c, for each *k*_*//*_ a Lorentzian curve centered at binding energy *x*_C_, with width *w*_L_ and initially arbitrary intensity at peak maximum (*A*) was plotted. The intensity at peak maximum were adjusted to results in the same integrated area for each Lorentzian function, the integral being numerically evaluated in the (${x_{\rm{C}}} - 5 \cdot {w_{\rm{L}}};{x_{\rm{C}}} + 5 \cdot {w_{\rm{L}}}$) energy range. For the gap region where two Lorentzian curves were extracted, the superimposition of the two curves was considered. The integral were calculated in the ($x_{\rm{C}}^{{\rm{Up}}} - 5 \cdot w_{\rm{L}}^{{\rm{Up}}};x_{\rm{C}}^{{\rm{Lo}}}{\rm{ + }}5 \cdot w_{\rm{L}}^{{\rm{Lo}}}$) binding energy range where $x_{\rm{C}}^{{\rm{Up}}}$ ($x_{\rm{C}}^{{\rm{Lo}}}$) and $w_{\rm{L}}^{{\rm{Up}}}$ ($w_{\rm{L}}^{{\rm{Lo}}}$) are the peak center (Lorentzian width) of H^U^ and H^L^ band components (Fig. 2b).

### Self-energy analysis

The self-energy Σ was defined as in ref. ^16^ i.e., *A(k*_*//*_ *, E)* ∝ ImΣ(*E*)/([*E-ε*_*k*_ *-*RealΣ(*E*)]^2^ + [ImΣ(*E*)^2^]) where *A(k*_*//*_ *, E)* is the quasiparticle spectral function, *ε*_*k*_ the non-interacting band dispersion. The real part RealΣ of the quasiparticle self-energy was extracted from HOMO-EDCs at various *k*_*//*_ by evaluating the differences between experimental and theoretical peak position. In standard ARUPS analysis, this quantity is generally extracted by comparing experimental and theoretical momentum distribution curves (MDCs). In the present case, however, the reduced HOMO band dispersion near Γ point (<10 meV) introduces large *k*_*//*_ broadening of the MDCs curve and large uncertain in the experimental HOMO peak position. The same reasons hinder a direct evaluation of the imaginary part ImΣ of the quasiparticle self-energy which reflects the *k*_*//*_ broadening of the experimental MDCs curves^16^.

### Estimation of the hole effective mass

The hole effective mass at HOMO edge was defined as m* = *ħ* ^*2*^/[d^2^E(k_*//*_ *)*/d^2^ *k*_*//(k//=0)*_)], where d^*2*^E(k_*//*_)/d^*2*^ *k*_*//(k//=0)*_ is the band curvature at Γ point (*k*_*//*_ = 0.000 Å^−1^). The band curvature was measured by parabolic fitting of the experimental data in the [0.000–0.070] Å^−1^ wa**v**e vector range, which reflects the band distortion produced by the kink. A value of 1.4 *m*_0_ < m* < 2.4 *m*_0_ was found, considering the binding energy and wave vector uncertain in the HOMO peak positions. Representative parabolic fitting curves for the m* upper and lower limit are reported in Fig. 2b. The same fitting method was applied to the theoretical band dispersion^18^ within the same wave vector range. A value of m* = 0.9 *m*_0_ was extracted which is consistent with the theoretical estimation in ref. ^18^ (0.9 *m*_0_) and in good agreement with previous reports (~0.8 *m*_0_)^10, 15^.

### Data availability

The data that support the findings of this study are available from the corresponding authors upon reasonable request.

## Electronic supplementary material

PDF SI

TPR
