# Supplementary material for: Hole-phonon coupling effect on the band dispersion of organic molecular semiconductors
Source: Nat Commun. 2017 Aug 2;8:173. doi: 10.1038/s41467-017-00241-z (PMC5539254; doi:10.1038/s41467-017-00241-z)
Supplement: Supplementary file 1 — PDF SI [file 41467_2017_241_MOESM1_ESM.pdf]

File name: Supplementary Information
Description: Supplementary figures, supplementary notes and supplementary references.

File name: Peer Review File
Description:

Supplementary Figures

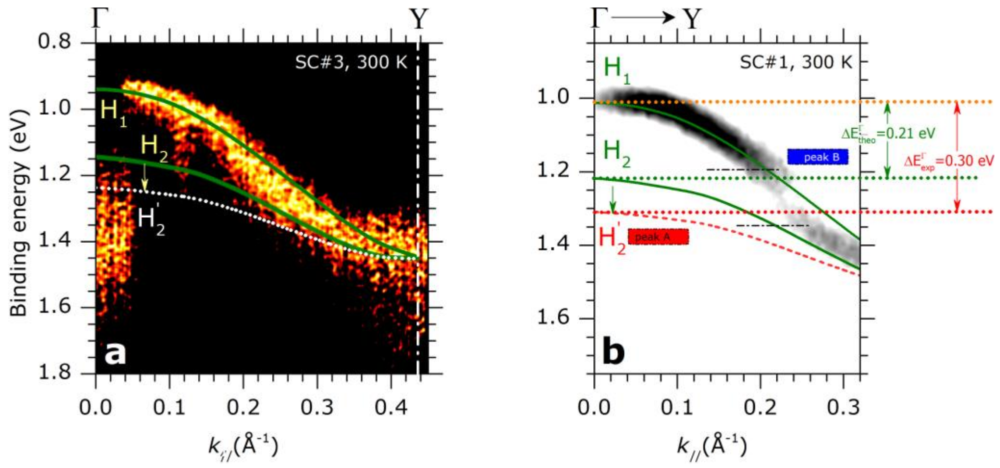

Supplementary Figure 1: Rubrene HOMO band dispersion. (a) HOMO-ARUPS intensity vs. $k_{//}$ as measured for a rubrene SC (SC#3) along ΓY direction (full range). The full range intensity map were obtained by combining two different ARUPS dataset acquired at 12° and 22° off normal conditions (see experimental geometry described in Fig. 1c of the main text). The photoemission intensities were normalized at the HOMO peak. Experimental data were compared with the DFT calculated band structure (green curves). Two HOMO bands (H_1 and H_2) are theoretically predicted according to the presence of two inequivalent rubrene molecules in the surface unit cell. Theoretical data were aligned at the HOMO peak position measured at the Γ point. The compressed band (H_2') is also reported to correctly reproduce the experimental energy separation between H_1 and H_2 band at Γ **(b)** Second derivative ARUPS intensity map ($-d^2E(k)/d^2k_{//}$) as obtained from ARPES data of SC#1. The data are the same as in Figure 2(b) of the main text. Dash dotted horizontal line define the gap boundaries. Theoretical data were aligned at the HOMO peak position measured at the Γ point. Note the difference between the theoretical (ΔE^{Γ}_{theo}=0.21 eV) and experimental (ΔE^{Γ}_{exp}=0.30 eV) energy separation between H_1 and H_2 band at Γ point. The compressed H_2 band dispersion (H'_2) is also plotted (see text for further details). The position of A and B band are also indicated.

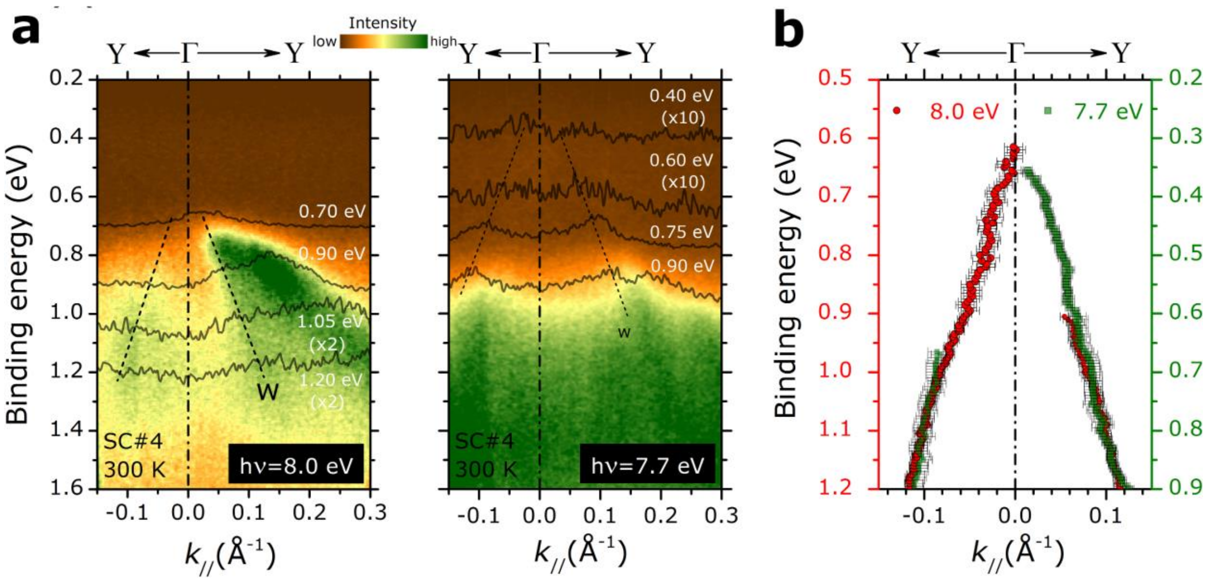

Supplementary Figure 2: Dispersion of *w* band. (a) ARUPS intensities *vs*. $k_{//}$ along the ΓY direction of rubrene single crystal SC#4 as acquired at hν=8.0 eV (left) and hν=7.7 eV (right) of photon energy. All data were acquired at 300 K. MDCs sample curves at different binding energy values were reported (black line). The energy dispersion of the *w* band is indicated by dash dotted lines as a guide for the eyes. (**b)** Experimental binding energy dispersion of the *w* band as extracted by the MDC curves from data in panel (**a)** at hν=8.0 eV (red circle, left scale) and hν=7.7 eV (green square, right scale). The binding energies of *w* peaks move according to photon energy changes (0.3 eV) i.e. the measured kinetic energies at *w* peaks remains constant at variance of the photon energy.

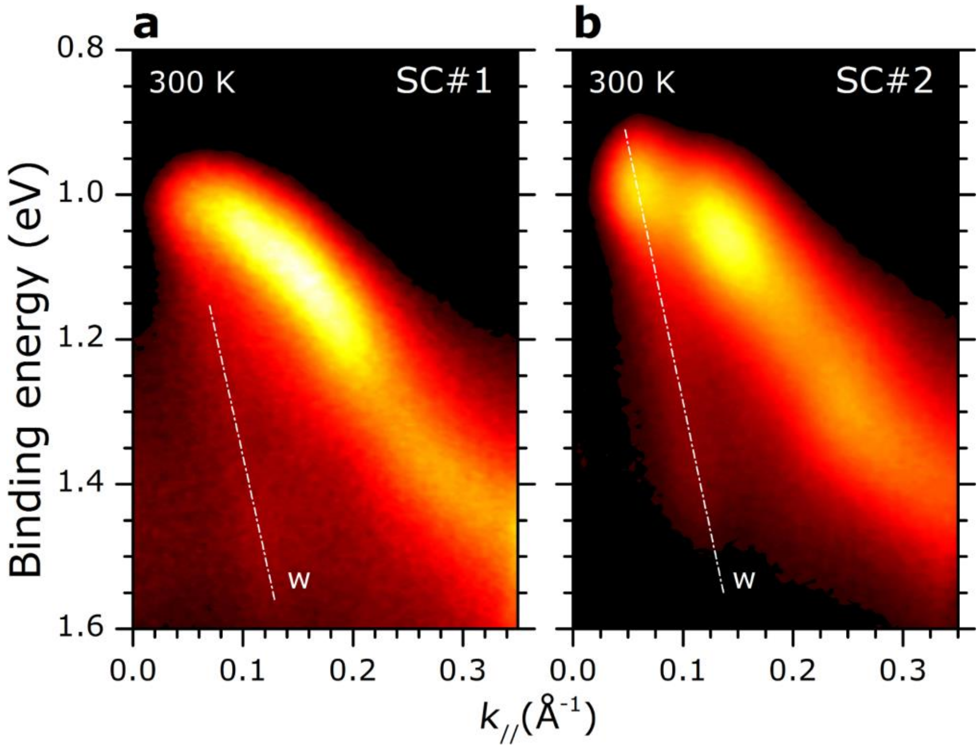

Supplementary Figure 3: HOMO intensity maps in different rubrene single crystals. Comparison between the ARUPS intensity vs. $k_{//}$ of SC#1 (a) and SC#2 (b) in the HOMO binding energy range. Both data were acquired along the ΓY direction at 300 K of sample temperature and reported in the same intensity scale range). Dash dotted line is a guideline for the *w* band. For both SCs a maximum of the HOMO band intensity is observed at around 0.15 $Å^{-1}$. At lower $k_{//}$, the HOMO band intensity of SC#1 rapidly vanishes towards the Γ point. In SC#2, after initial decreasing, the ARPES signal of the HOMO band apparently increases due to its superimposition with the largely dispersive HOMO band. This apparent intensity modulation of the HOMO band along the high symmetry direction is absent in SC#2 due to the lower intensity of the *w* band.

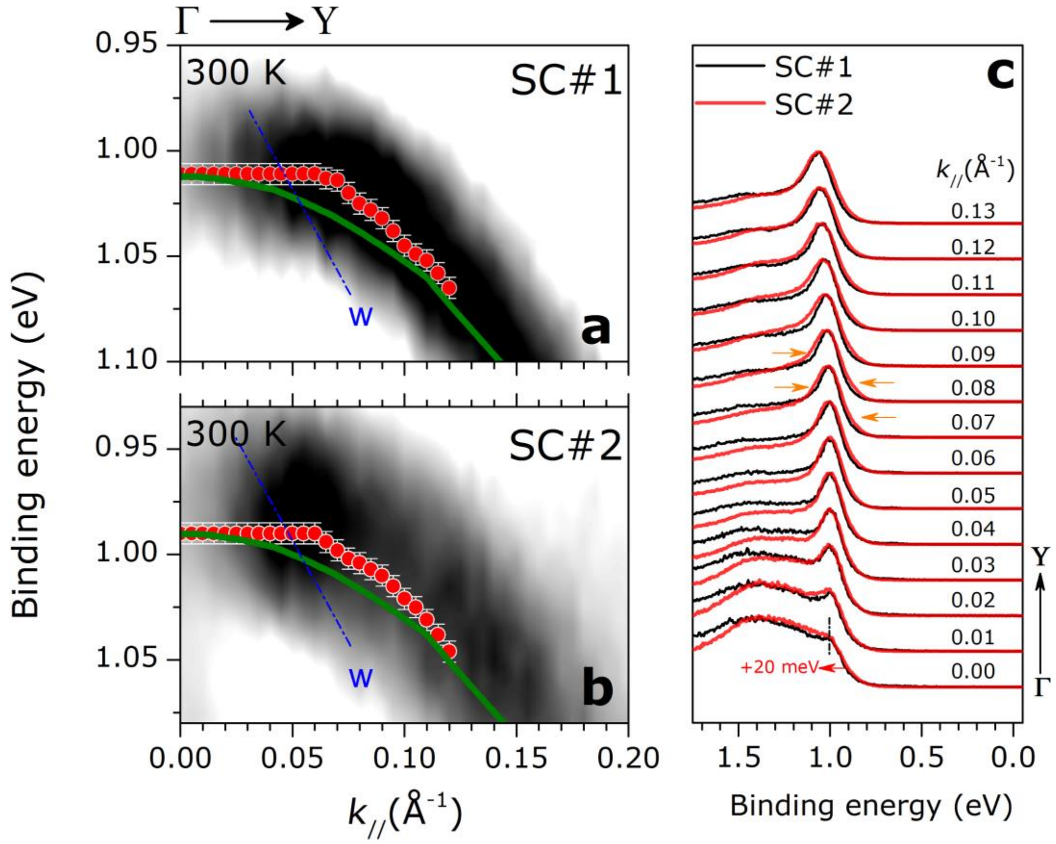

Supplementary Figure 4: Second derivative intensity maps in different rubrene single crystals. Second derivative ARUPS intensity map of SC#1 **(a)** and SC#2 **(b)** along the ΓY direction. **(c)** Comparison between EDCs of SC#1 and SC#2 as obtained along the ΓY direction. Binding energy values are aligned at the SC#1 HOMO peak position at Γ. For each EDCs, the intensity are normalized at the peak maximum.

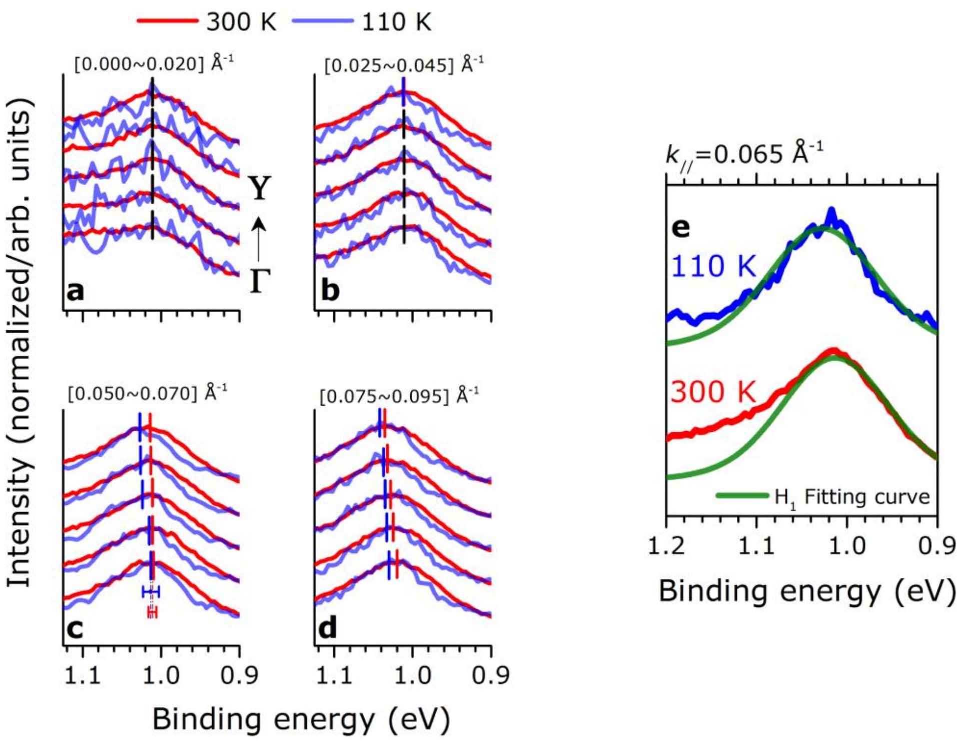

Supplementary Figure 5: Impact of the sample temperature on the HOMO EDCs (a)-(d). Energy distribution curves (EDCs) of rubrene SC#1 as obtained in the [0.000~0.095] wavevector ($k_{//}$) range at 300 K (red curves) and 110 K (blue curve). Lower (upper) curves corresponds to the minimum (maximum) value of the indicated wavevector range. The data were plotted after Shirley background removal and the intensity normalized to the peak maximum. The position of the H_1 peak as obtained by fit (see Methods section of the main text for details) is indicated by red (300 K) and blue (110 K) vertical bar. When the peak position of 300 K and 110 K EDCs coincides) the peak position is marked by black vertical bars. Error band in peak position determination are indicated in panel (c) as horizontal bars. Despite a larger signal-to-noise ratio in the low temperature data as due to the lower photon flux during 110 K data acquisition (i.e. for reducing possible sample charging, see main text for details) a clear shift of the peak centroid towards high binding energy is observed at decreasing of the sample temperature in the ~[0.06~0.09] $Å^{-1}$ wavevector range. This corresponds to the quenching of the kink structure in the H_1 band dispersion. (e) Representative peak fitting of the EDCs in panel **(a)-(d).**

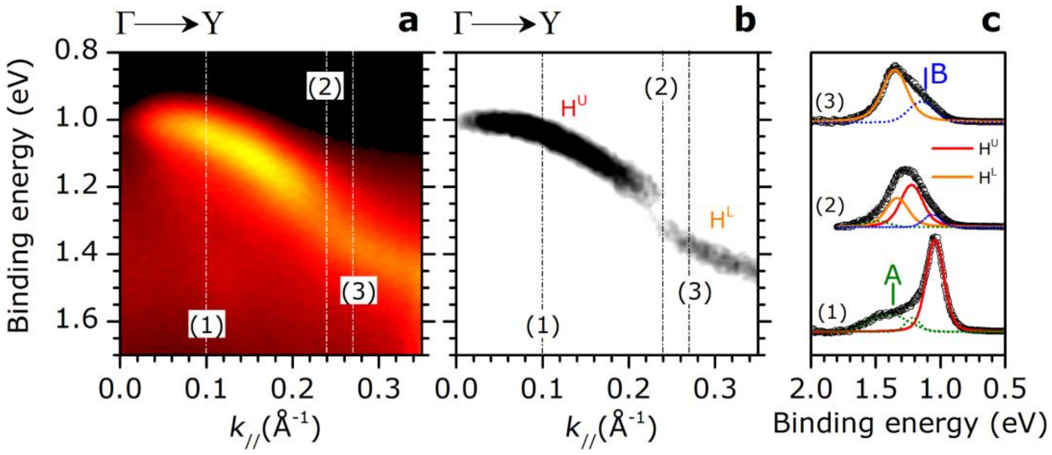

Supplementary Figure 6: HOMO peak fitting procedure. **(a)** ARUPS intensities vs. $k_{//}$ along the ΓY direction of rubrene single crystal SC#1 at 300 K. **(b)** Second derivative ARUPS intensity map as obtained from data in panel (a). **(c)** HOMO peak fitting of the EDCs curves (1) (2) and (3) as indicated in panel (a).

Supplementary Note 1: Rubrene Theoretical HOMO bands dispersion.

According to band structure DFT calculations [1] two dispersive HOMO derived band H_1, H_2 are predicted along the ΓY direction of rubrene single crystal [Supplementary Figure 1(a). In previous ARUPS investigations of rubrene single crystal, two HOMO derived subbands (H_1, H_2) were consistently reported as resulting from the presence of two inequivalent molecules per unit cell [2, 3] The UPS intensity of the higher binding energy band (H_2) is generally much lower than that of the H_1 band and it is clearly visible mainly in the nearby of the Γ point. These differences in peak intensity and angular dependence were observed in a wide range of photon energy (20~40 eV) as well in correspondence of various UPS experimental geometry. Similar differences in the HOMO subbands intensity and widths are also observed in ARPES measurements for a wide range of organic single crystal and thin films [2,3]. These differences can be tentatively ascribed to photoemission matrix element effect reflecting the different spatial distribution of the H_1 and H_2 wavefunctions. In case of isolated or weakly interacting molecules the angular distribution of the photoelectron intensity at variance of the photon energy is well theoretically understood and it is closely related to the symmetry of the molecular [4]. For 3D solids the situation is more complex as mainly due to the spatial delocalization of the HOMO wavefunctions and a complete understanding of the photoelectron angular distribution of UPS intensity has yet still to be provided. A detailed understanding of this intensity difference is beyond the current theoretical understanding of the photoemission process in organic solids and certainly beyond the scope of the present contribution.

Moreover, a direct comparison of the experimental band dispersion with the theoretical band structure may suggest the possible identification of the H^U and H^L with the H_1 and H_2 theoretical bands, their intensity being rapidly modulated by possible photoemission matrix effects. This identification can be excluded of the basis as discussed in the following.

In ARUPS investigation of single crystal materials, rapid spectral intensity changes at variance of wavevectors component are generally observed around the high symmetry points of the Surface Brillouin Zone (SBZ). This intensity modulation can be ascribed to matrix element effect depending in turn on the wavefuntions symmetry, incident light polarization, the photon energy, and the experimental geometry. In the present case of rubrene single crystal, the suggested intensity modulation of the H_1 and H_2 band would occur within the SBZ where no high symmetry points, by definition, exist. This makes the proposed identification of the H^L band with H_2 band quite unlikely.

In principle, additional high symmetry points can be introduced by in plane crystal reconstruction in the bulk and/or surface region. However change in the in-plane lattice periodicity of the bulk region are not supported any reported X-ray diffraction data. Moreover, high-resolution frequency modulation atomic force microscopy investigation of rubrene single crystal excluded the occurrence of any surface reconstructions [10].

At Γ point, the experimental energy separation between the H_1 and H_2 band is ~0.30 eV as obtained by previous ARPES investigation [2,3] with respect to a theoretical value of ~0.2 eV [Supplementary Figure 1(b)]. In this context, while the theoretical calculations reproduce quite well the H_1 experimental band dispersion, the bandwidth of the H_2 band is slightly overestimated. In order to reproduce the energy separation at Γ point the H_2 theoretical band dispersion must be ~30% "compressed" along the binding energy axis. This results is a further deviation of the H_2 band with respect to the experimentally measured "lower" (H^L) subband of rubrene single crystal. In this context the identification of the H^L with H_2 band as modulated by cross section effects (if any) is unlikely.

In addition to the above considerations we note that, near the Γ point, the energy of the H_2 is located very closely to the rubrene vibrational energy "cut" [Supplementary Figure 1(b)]. A similar situation was reported for single crystalline Pn where hole-phonon coupling led to increased energy separation between the HOMO sub-bands and reduction of the HOMO bandwidths [9]. In this context, the energy positions of A peak may reflect the increase of the energy separation between H_1 and H_2 sub-bands at Γ point as resulting from hole coupling with *intra*molecular vibrations. The superimposition with secondary electron background and *w* band may hinder the detail of the A(H_2) band dispersion along ΓY direction. The band B is similarly located close to the intersection with vibrational energy [Supplementary Figure 1(b)]. These observations suggest the origin of A and B in the modification of the HOMO quasiparticle spectral function as resulting from coupling with *intra*molecular vibrations. However, further theoretical studies are required to clarify this point.

Supplementary Note 2: Origin on *w* band.

In order to clarify the origin of *w* band, the ARUPS spectra of a rubrene sample (SC#4) were carefully measured close to Γ point and acquired at variance of the energy of the incident UV light. The corresponding intensity map in the energy momentum/space as obtained at hν=8.0 eV and hν=7.7 eV are reported in Supplementary Figure 2. The *w* peak positions were evaluated from the corresponding momentum distribution curves (MDCs). Representative MDCs curves are shown in Supplementary Figure 2(a). The *w* band dispersions are reported in Supplementary Figure 2(b), as extracted from the MDCs curves. Interestingly, on the binding energy scale the *w* band position moves according to photon energy changes (0.3 eV) i.e. the kinetic energies at *w* peaks remains constant at variance of the photon energy. This peculiar behaviour was previously reported in photoemission investigation on HOPG and single crystal graphite [5,6] and was related to the high density states in the (unoccupied) conduction band of graphite. The photoelectrons excited from the valence bands undergo a series of electron scattering and produces cascaded secondary electrons. These electrons can make transitions to the unoccupied states of graphite from which they escape into the vacuum without any memory of the initial energy of the occupied states resulting in the constant kinetic energy band dispersions [5]. The intensity

of these states depends on the quality and cleanliness of the graphite surface, being rapidly quenched by the presence of surface structural disorder and the adsorption of molecular species [6]. Consistently, the intensity of *w* band in rubrene single crystal is generally rather weak in comparison with other rubrene spectral features and it strongly depends of the measured sample (see comparison between SC#1 and SC#2 in Supplementary Figure 3). This reflects the different quality of the surface regions in term of amount of surface contaminants and/or structural defects originating by surface photo oxidation process [7]. According to the above consideration, the *w* band reflects the density of states and the band dispersions of unoccupied states of rubrene single crystal. In order to get rid of the surface quality problem, a series of ARUPS measurements on UHV cleaved rubrene single crystals are planned. This is expected to allow a better evaluation of the *w* band dispersion and therefore a more systematic ARUPS mapping of unoccupied band structure of the organic single crystal**.**

Finally, in both crystals SC#1 and SC#2 a clear kink structure is observed near the Γ point, thus indicating that the superimposition with *w* band does not significantly affect the measured HOMO band dispersion [Supplementary Figures 4(a),(b)]. This can be qualitatively explained considering the higher "slope" of the *w* band dispersion with respect to the HOMO band in the nearby of the Γ point. In this context, the superimposition with the *w* band only results in an HOMO peak broadening, as indicated by arrow in Figure 4(c) in a limited momentum range without affecting the position of the HOMO peak maximum. Note that the broadening effect is larger in the case of SC#2 crystal as consistent with the higher intensity of the *w* band.

Supplementary Note 3: Local hole-phonon coupling effect on the HOMO band dispersion

In nearly isolated organic molecules as in gas phase or organic thin films with negligible intermolecular lateral interaction, the non-dispersive HOMO band consists of several components with nearly equal energy separations [8]. By considering the coupling with a single vibrational mode this energy separation (Ω_0, typically $\gtrsim$100 meV) may be identified with the intramolecular vibration energy while the relative intensities of the HOMO components reflects the strength of the hole-phonon coupling [9]. In molecular crystal, where larger HOMO bandwidth is expected, the situation is more complicated and the problem was recently considered by Ciuchi *et al*. [9]. In particular, the HOMO spectral function was directly calculated, which reflects the energy and momentum dependence of the ARUPS spectral intensity. When $W \gtrsim \Omega_0$, the vibrational structure characteristic of the molecular spectra is replaced by "cuts" in the energy band dispersion and the HOMO is split in a series of sub bands separated by a sizable vertical band gap of ~Ω_0 (See Figure 1 of Supplementary Reference 9).

Supplementary References.

[1] Yanagisawa, S., Morikawa, Y., & Schindlmayr, A., HOMO band dispersion of crystalline rubrene: Effects of self-energy corrections within the GW approximation, *Phys. Rev. B* **88**, 115438-115444 (2013).

[2] Machida, S. I., Nakayama, Y., Duhm, S., Xin, Q., Funakoshi, A., Ogawa, N., Kera, S., Ueno, N.,& Ishii, H., Highest-occupied-molecular-orbital band dispersion of rubrene single crystals as observed by angle-resolved ultraviolet photoelectron spectroscopy, *Phys. Rev. Lett.* **104**, 156401-156404 (2010)

[3] Nakayama, Y., Uragami, Y., Machida, S., Koswattage, K., Yoshimura, D. , Setoyama, H. , Okajima, T. , Mase, K. & Ishii, H., Full Picture of Valence Band Structure of Rubrene Single Crystals Probed by Angle-Resolved and Excitation-Energy-Dependent Photoelectron Spectroscopy, *Appl. Phys. Express* **5**, 111601-111604 (2012)

[4] Wießner, M., Hauschild, D., Sauer, C., Feyer, V., Schöll, A., & F. Reinert, F., Complete determination of molecular orbitals by measurement of phase symmetry and electron density, *Nat. Comm*. **5**, 4156-4161(2014).

[5] Mahatha, S. K. , Menon, K.S.R. , & Balasubramanian, T., Unoccupied electronic structure of graphite probed by angle-resolved photoemission spectroscopy, *Phys. Rev. B* **84**,113106-113109 (2011).

[6] Yamane, H. , Nagamatsu, S. , Fukagawa, H., Kera, S. , Friedlein, R. , Okudaira, K. K. &Ueno,N., *Phys. Rev. B* **72**, 153412-153415 (2005).

[7] Mastrogiovanni, D. D. T., Mayer, J., Wan, A. S., Vishnyakov, A., Neimark, A.V., Podzorov, V., Feldman, L. C. & Garfunkel, E., *Sci. Rep.* **4**, 4753-4758 (2014).

[8] Ueno, N. & Kera, S. Electron spectroscopy of functional organic thin films: Deep insights into valence electronic structure in relation to charge transport property, *Prog. Surf. Sci.* 83, 490-557 (2008).

[9] Ciuchi, S. & Fratini, S., Band dispersion and electronic lifetimes in crystalline organic semiconductors, *Phys. Rev. Lett.* **106**, 166403-166406 (2011).

[10] Minato, T., Aoki, H., Fukidome,H.,Wagner,T., & Itaya, K., High-resolution molecular images of rubrene single crystals obtained by frequency modulation atomic force microscopy, *Applied Physics Letter*s **95**, 093302-0903304 (2009)

[11] Ciuchi, S., Hatch, R. C., Hochst, H., Faber, C., Blase, X., & Fratini, S., Molecular Fingerprints in the Electronic Properties of Crystalline Organic Semiconductors: From Experiment to Theory, *Phys. Rev. Lett*. **108**, 256401-256405 (2012).
