# Supplementary material for: Hole-phonon coupling effect on the band dispersion of organic molecular semiconductors
Source: Nat Commun. 2017 Aug 2;8:173. doi: 10.1038/s41467-017-00241-z (PMC5539254; doi:10.1038/s41467-017-00241-z)
Supplement: Supplementary file 2 — TPR [file 41467_2017_241_MOESM2_ESM.pdf]

Reviewers' comments:

Reviewer #1 (Remarks to the Author):

In this article the authors present a study of the electronic band structure of rubrene single crystals by angle-resolved photoelectron spectroscopy (ARPES).
They find a strongly dispersing band derived from the rubrene highest occupied molecular orbital (HOMO), which shows the subtle influence of the coupling to lattice phonons and molecular vibrations.

The present work is the result of Prof. Kera's group's systematic and successful efforts in extending their world leading expertise in the analysis of epitaxial molecular layers by high-resolution photoelectron spectroscopy to molecular single crystals. While a lot of very important knowledge has been derived from work on thin films (see e.g. Ref. 4 and 20 of the manuscript), single crystals can be regarded as a more accurate reference for applications in several respects, due to the perfect long range order, very low level of defects, and the resulting delocalized states. Technically, ARPES experiments with such single crystals are not an easy task, since crystal growth, handling and surface preparation in the UHV, as well as charging and radiation damage during the experiments are mayor problems. However, the Kera group and collaborators have solved these problems already a while ago, which lead to the first observation of a band dispersion in organic single crystals in 2010 (see Ref. 22 of the manuscript).

While the dispersion has been demonstrated before, the effect of the coupling to vibrations/phonons is in the focus of the present manuscript and can be analysed in detail due to the further increased accuracy in the experimental data. This coupling is a novel observation and confirms previous theoretical predictions.

The presented data is of exceptional quality and the results are supported well by this data. The scientific impact of this work is high, since it not only demonstrates, that concepts usually applied in inorganic materials can also be applied to molecular solids, but it is also of crucial importance for the comprehensive understanding of charge transport phenomena in molecular materials. Knowledge of the latter is of paramount importance for applications of molecular materials, since it is a prerequisite for an optimization of such systems in organic devices. I therefore support publication of this manuscript. It is furthermore of interest to a broad readership which is adequately addressed by Nature Communications.

However, prior to publication, the authors should consider the following points:

- One of the central weaknesses of the present work is that the experiment does reproduce the effect of vibronic coupling as predicted by the calculations, but not the much more prominent splitting of the HOMO band into H1 and H2. Although the authors comment on this issue in the supplement, they should give a concise explanation in the main text, why they think the H2-band is missing in their data. On first glance, the experimental data fits very nicely to H1 and small k and to H2 at large k-values. Can an explanation along this line, maybe due to certain cross section effects, be ruled out?

- According to the temperature dependent experimental data, coupling to intramolecular vibrations occurs in the photoemission final state, while the coupling to intermolecular phonons is an initial state effect which can be frozen out by cooling to 110K. In my understanding, the former is thus a coupling between the photo-hole and a molecular vibration, the latter a coupling between the electrons in the delocalized HOMO-band and lattice vibrations (or phonons) already in the initial state, and not a result of "hole-phonon coupling", as cited from the title. If this is correct, it would be helpful for the reader if

the authors' discussed this more precisely.

Minor comments:
- Although generally written clearly, the entire manuscript would benefit from a further round of editing. In particular, there are several grammatical mistakes and missing articles.
- Line 133ff: It is not clear, how the "Lorentzian component" plotted in Fig. 2c was derived from the fit to a Voigt functions. I understand that the widths of the Lorentzian and Gaussian can be separated, the "Lorentzian component" used by the author seems to be something like and area or spectroscopic weight of the Lorentzian. How should this be derived from the Voigt function, which is a convolution of a normalized Lorentzian and Gaussian?
- Line 226: "intramolecular" must be "intermolecular"
- Line 440: "300 K" must be "110 K"
- Line 441: "… as a function of sample temperature." should obviously be "… at 110 K.", since there is no temperature dependent data in Fig. 3b.
- In Fig. 1-3 the colour code must be given.
- Fig. 3c does not share the y-axis with Fig. 3a and b. The y-axis in this case is "intensity" or "normalized intensity" and must be denominated accordingly.
- Line 192 ff: I don't understand the sentence: "At 300 K, a peak at ~15 meV from the top of the HOMO band (HΓ) is visible, which corresponds to the energy of the coupled intermolecular vibrations [9]."

Reviewer #2 (Remarks to the Author):

The current research in this topic is well framed in the introduction and the motivation is strong: understanding experimentally the coupling of intra- and inter-molecular motions with the hole. The experiments are highly non-trivial.

Before giving some detailed comment maybe it is useful for the authors to say that I have the impression that the results are fairly inconclusive possibly because the resolution of the data and the lack of directly comparable theory. I think there is a tendency to find what one wants to find, i.e. signature of coupling with high frequency modes and low frequency modes. However I find neither of them convincing.

It is far from obvious that there is a gap in figure 1a (it could be artefact of the intensity diminishing). If there is, its amplitude can be anything between 40 and 200 meV with this resolution. Its origin remains unclear. For once, it doesn't look like the one predicted in ref.[11]. Secondly the high frequency electron-phonon coupling for rubrene are known [Adv. Mater. 17, 1072–1074 (2005)]. These couplings are not too strong and there is a range of modes that contribute in the range 100-200 meV. I find it hard to imagine these result in a gap in the measured ARUP explainable with a single mode. Certainly this deserves a better explanation.

An analogy with ref.[9] is used to explain the possible role of intramolecular modes. The material is so different that this analogy is not clear at all. It was part of the motivation of this work to investigate a system where traditional band transport concepts fail but the same ideas are stretched to explain the results in terms of increased effective mass. The raw results do not show clear temperature dependent effect and the data presented in Figure 4 are not very clear to me. It would be much better to present a model that reproduce directly the experimental results rather than using the model to extract

information that combine experimental data and assumptions of the model.

At the moment I don't know if clarifications and improved modelling can bring this manuscript to the standard of Nature Comm.

Reviewer #3 (Remarks to the Author):

Measurements of the HOMO band dispersion of rubrene single crystals have been quite often reported in the literature (several by some of these authors). The data here is however of very high quality and resolution allowing hitherto unseen fine structure to become apparent. These bear a strong resemblance to phenomena proposed, from theoretical deliberations in ref 11, to arise from hole-phonon coupling and the data was analysed accordingly.The regions critical to their interpretation are the gap at ~0.22 A-1 and a kink at around 0.065A-1 assigned to the effects of intra- and intermolecular vibrations, repectively. Because of it significance to charge transport in organics I believe the work warrants publication if the following points are considered.

What I find puzzling is that two bands are expected but "no experimental evidence" for the 2nd band (H2) was found. Vollmer et.al (J.El.Spec.185(2012)55) do seem to see both bands with the expected separation near gamma of ~0.25 eV. Without an understanding of why the H2 is missing the gap opening argument is weakened. For instance, it might be argued that in the first half of the Brillouin zone the first band is strong while in the 2nd half the H2 dominates – then the observation would not be gap opening in a single band.

The disappearance of the kink in going to low temperatures is critical to this work however it is not compellingly presented in the data. EDCs at 300 and 110K in the region of the kink (~0.065A-1) need to be added in Fig3c so that the reader may judge whether the kink has indeed disappeared at 110K.
Minor points:
The colour chosen for the data points in the inset of of fig. 3b make them almost invisible.
The theoretical bands should be smooth and not kinked as they appear in the figures.
The schematic of the real space unit cell in Fig 1a is too small to be useful – it should be also added to supplementary information with a reasonable size.
Fig 1d is almost redundant- if they have the data it should be extended to include the BZ boundary (Y)

REVIEWERS' COMMENTS:

Reviewer #1 (Remarks to the Author):

In the revised version of their mansucript the authors have taken all critics raised in my original report into account. They have taken care to correct all minor mistakes and thoroughly replied to all comments. I consequently support publication of this manuscript in Nature Communications without further revision.

Reviewer #2 (Remarks to the Author):

In essence this paper reports top quality experimental results with some plausible but, to some extent, non-quantitative explanation of the observations. On balance I think this work should be published on Nature Communications possibly with the inclusion in the main manuscript of some of the comments made by the authors in the response to the referees about the lack of quantitative theory to explain these experiments.

Essentially two new features are observed one of which temperature dependent in the 100-300 K range. It would be nice if the temperature in-dependent effect is due to high frequency phonons and the temperature dependent one is due to low frequency phonons, and this is their main point. The latter is certanly true, and the former it possibly true.

The observation itself does not allow labelling the hole-phonon coupling as local or non-local, this derives from calculations showing the the non-local coupling is important at low frequency and the local coupling is important at high frequency.

Even less based on the observation is the labelling of the low frequency modes effect as "inter-molecular" mode. The modes can be very well intramolecular and affect the transfer integral, i.e. be non-local.

My recomendation is therefore to publish this work on NC with few additional sentences highlighting what are the most speculative aspects of this paper and those requiring more quantitiative theoretical work. It should become clear that this paper represents an important step forward rather than achieveing a complete understanding of the hole-phonon coupling effect.

Reviewer #3 (Remarks to the Author):

The responses are just satisfactory. The addition of Figure S3 was necessary- I guess some readers will believe the kink removal others not - I tend to be a believer. Interpretations can change whats important is that the data stands the test of time- and I feel the community will not get better data to work with for a number of years. All referees were disturbed by the missing H2 band - the answer of matrix effects is of course true but says nothing- the reason I wanted to see the real space orientation was so I could think about reasons for H2s vanishing intensity (and for that one needs to know the experimental geometry, including the molecules orientations). Your Response "we believe that the size of the figure is suitable for reader" was therefore extremely irritating and clearly wrong as I am a reader!

Reply to reviewer #1

Comment #1: In this article the authors present a study of the electronic band structure of rubrene single crystals by angle-resolved photoelectron spectroscopy (ARPES). They find a strongly dispersing band derived from the rubrene highest occupied molecular orbital (HOMO), which shows the subtle influence of the coupling to lattice phonons and molecular vibrations. The present work is the result of Prof. Kera's group's systematic and successful efforts in extending their world leading expertise in the analysis of epitaxial molecular layers by high-resolution photoelectron spectroscopy to molecular single crystals. While a lot of very important knowledge has been derived from work on thin films (see e.g. Ref. 4 and 20 of the manuscript), single crystals can be regarded as a more accurate reference for applications in several respects, due to the perfect long range order, very low level of defects, and the resulting delocalized states. Technically, ARPES experiments with such single crystals are not an easy task, since crystal growth, handling and surface preparation in the UHV, as well as charging and radiation damage during the experiments are mayor problems. However, the Kera group and collaborators have solved these problems already a while ago, which lead to the first observation of a band dispersion in organic single crystals in 2010 (see Ref. 22 of the manuscript).

While the dispersion has been demonstrated before, the effect of the coupling to vibrations/phonons is in the focus of the present manuscript and can be analysed in detail due to the further increased accuracy in the experimental data. This coupling is a novel observation and confirms previous theoretical predictions.

The presented data is of exceptional quality and the results are supported well by this data. The scientific impact of this work is high, since it not only demonstrates, that concepts usually applied in inorganic materials can also be applied to molecular solids, but it is also of crucial importance for the comprehensive understanding of charge transport phenomena in molecular materials. Knowledge of the latter is of paramount importance for applications of molecular materials, since it is a prerequisite for an optimization of such systems in organic devices. I therefore support publication of this manuscript. It is furthermore of interest to a broad readership which is adequately addressed by Nature Communications.

Reply #1: We thank the referee for appreciating our experimental efforts in providing high quality ARUPS data of organic single crystal.

C#2: One of the central weaknesses of the present work is that the experiment does reproduce the effect of vibrionic coupling as predicted by the calculations, but not the much more prominent splitting of the HOMO band into H1 and H2. Although the authors comment on this issue in the supplement, they should give a concise explanation in the main text, why they think the H2-band is missing in their data. On first glance, the experimental data fits very nicely to H1 and small k and to H2 at large k-values. Can an explanation along this line, maybe due to certain cross section effects, be ruled out?

R#2: In organic systems with two inequivalent molecules per unit cell, the HOMO band is split into two subbands. UPS peak intensity and width of the two HOMO-derived bands is generally different [See Ref. 4 in the main text]. These differences can be tentatively ascribed to matrix element effect in the photoemission process as related to the symmetry of the HOMO wavefunctions, energy and polarization of incoming photons and emission direction of the photoelectrons [See Ref. 4 in the main text]. In case of single or weakly interacting molecules the angular distribution of the photoelectron intensity at variance of the photon energy is well theoretically understood [Nat. Comm. **5**, 4156 (2014)] and it is closely related to the symmetry of the molecular orbitals in the nearly isolated molecules. For organic solids the situation is more complex as mainly due to the spatial delocalization of the HOMO wavefunctions and a complete understanding of the photoelectron angular distribution of UPS intensity has yet still to be provided.

In previous ARUPS investigation of rubrene single crystal, two HOMO derived subbands (H_1, H_2) were reported as resulting from the presence of two inequivalent molecules per unit cell [Phys. Rev. Lett 104, 156401 (2010), Appl. Phys. Express 5, 111601 (2012)]. **The observed UPS measured intensity of the higher binding energy band (H_2) is generally lower (<40%) than that of the H_1 band and it is clearly visible mainly in the nearby of the Γ point.** These differences were observed in a wide range of photon energy (20~40 eV) as well in correspondence of various UPS experimental geometry. As mentioned above the differences can be ascribed to photoemission matrix effect reflecting the different spatial distribution of the H_1 and H_2 wavefunctions. A detailed understanding of this intensity

difference is beyond the current theoretical understanding of the photoemission process in organic solids and therefore certainly beyond the scope of the present contribution.

C#3: One of the central weaknesses of the present work is that the experiment does reproduce the effect of vibronic coupling as predicted by the calculations, but not the much more prominent splitting of the HOMO band into H1 and H2. Although the authors comment on this issue in the supplement [no splitting of the HOMO band into H1 and H2], they should give a concise explanation in the main text, why they think the H2-band is missing in their data. On first glance, the experimental data fits very nicely to H1 and small k and to H2 at large k-values. Can an explanation along this line, maybe due to certain cross section effects, be ruled out?

R#3: The identification of upper H^U and lower H^L experimental band with the H_1 and H_2 band, respectively, with their intensity modulated by cross matrix effects can be excluded on the basis of the following arguments:

(a) In ARUPS investigation of single crystal materials, rapid spectral intensity changes at variance of wavevectors component are generally observed around the high symmetry points of the Surface Brillouin Zone (SBZ). This intensity modulation can be ascribed to photoemission matrix element effect depending in turn on (i) the wavefuntions symmetry, (ii) incident light polarization, (iii) the photon energy, and (iv) the experimental geometry. In the present case of rubrene single crystal, the intensity modulation, which the reviewer suggested to be related to the H_1 and H_2 band, occurs within the SBZ where no high symmetry point, by definition, exists. This makes the identification of the H^L band with H_2 band proposed by the reviewer quite unlikely. In principle, additional high symmetry points can be introduced by in plane crystal reconstruction in the bulk and/or surface region. However change in the in-plane lattice periodicity of the bulk region is not supported any reported X-ray diffraction data. Moreover, high-resolution atomic force microscopy (AFM) investigation of rubrene single crystal excluded the occurrence of any surface reconstructions [Applied Physics Letters 95, 093302 (2009)]

(b) The band structure calculations predict the existence of two dispersive bands along the ΓY direction of rubrene single crystal. This is confirmed by previous ARUPS reports on rubrene single crystal [Phys. Rev. Lett 104, 156401 (2010), Appl. Phys.

Express 5, 111601 (2012)]. At Γ point the experimental energy separation between the H_1 and H_2 band is ~0.30 eV [Appl. Phys. Express 5, 111601 (2012)] with respect to a theoretical value of ~0.2 eV. In this context, while the theoretical calculations reproduce quite well the H_1 experimental band dispersion, the bandwidth of the H_2 band is slightly overestimated. In order to reproduce the energy separation at Γ point the H_2 theoretical band dispersion must be ~30% compressed along the binding energy axis. This result is a further deviation of the H_2 band with respect to the experimentally measured "lower" (H^L) subband of rubrene single crystal. In this context the identification of the H^L with H_2 band as modulated by cross section effects (if any) is unlikely.

(c) It is worth to note in passing that the larger energy separation between observed HOMO derived sub-bands, a and b, with respect to the theoretical predictions (see refs. 16 and 17) were discussed by Ciuchi et al. (see ref. S4 in SM) for pentacene single crystal case as a consequence of the hole coupling with the intramolecular vibrations.

The following sentence was added to the main text (page 6, line 138 of the revised manuscript)

"The data reported in Fig. 2b may suggest a possible identification of the H^U and H^L subbands with H_1 and H_2 theoretical band respectively, their intensity being suddenly modulated by photoemission matrix element effect. This possibility is excluded, as discussed in section 2 of the SM."

The Supplemental Materials were modified accordingly to include the above discussion. Due to its length, we preferred to kept this discussion in the supplemental materials, as its insertion in the main text might make the present results unclear.

C#4: According to the temperature dependent experimental data, coupling to intramolecular vibrations occurs in the photoemission final state, while the coupling to intermolecular phonons is an initial state effect which can be frozen out by cooling to 110K. In my understanding, the former is thus a coupling between the photo-hole and a molecular vibration, the latter a coupling between the electrons in the delocalized HOMO-band and lattice vibrations (or phonons) already in the initial state, and not a result of "hole-phonon coupling", as cited from

the title. If this is correct, it would be helpful for the reader if the authors' discussed this more precisely.

R#4: Photoelectron excitation reflects (i) intrinsic coupling with intra- and intermolecular vibrations as demonstrated by the present observation of quasiparticle states and (ii) extrinsic coupling such as energy gain/loss events upon photoelectron scattering during escape to vacuum. The probability of the latter case is very small and could be negligible in the present discussion, while the former is very important not only to study photoionization of organic molecular solids but also to reveal their charge transport properties.

Electron-phonon coupling occurs both in the initial state (charge neutral state) and in the final state (ionized state). The photoelectron spectroscopy monitors the one-hole final state, which is described by a one-hole bound state (such as HOMO with one hole) and a photoelectron in continuum state, hence the term of "hole-phonon coupling" is used to describe the impact of any electron-phonon coupling at the one-hole final state in the present manuscript. In a simple approximation, the impact of electron-phonon coupling at the initial state may be considered to appear in the observed spectrum as so-called thermal broadening of energy levels because of thermal excitations of phonons. However quantum-mechanical description for the photoionization tells us that the photoelectron intensity (current) is given by absolute value of a correct final state wavefunction (one photoelectron state and one hole in a bound state with relaxed electrons and nucleus), in which we cannot discuss electron-phonon coupling effects in the charge-neutral initial state separately. From strict view point, we thus think that the idea of the reviewer is not correct. In this paper, therefore, we wanted to not overwrite the discussion of the temperature dependence that is an important target of theoretical studies. In photoelectron spectroscopy, furthermore, effects of the electron-phonon coupling depend on the time scale of relaxation effects after photohole creation [Čápek and Silinsh, Chem. Phys. 200, 300 (1995)], and should appear in the spectra depending on the incidence photon energy [namely on the speed (kinetic energy) of the traveling photoelectron, or say on the measurement time dependence]. These issues are not well understood theoretically at present. Such a time-dependent relaxation effect can also give broadening in the spectra. Therefore we want to be honest about such insufficient understanding of photoelectron spectroscopy of organic molecular crystals, and in this revised paper we do not want to over discuss these issues including temperature broadening

due to thermal excitation of phonons. We deeply hope understanding of these points by the reviewer.

In relation to the contribution of the above-discussed measurement time of the UPS, as in the theoretical description by Ciuchi et al. (see Eq. 1 of ref. 11 of the main text) both intermolecular and intramolecular coupling terms can be introduced in the Hamiltonian of the system. The calculation is performed to simulate the spectrum feature under nearly-full computation on the electronic states for the fully-relaxed polaron state of the one-hole final state, which would correspond to the use of sudden approximation on the ionization of time dependent relaxation phenomena.

In our experiments, UPS uses very low-photon energy, and thus its measurement time is much longer than usual He I UPS, which would have larger opportunity to observe 'more' relaxed polaron after photoionization (not only the high speed electron relaxation effects but also slower relaxation of nucleus positions upon ionization with smaller-frequency crystal phonons/molecular-based phonons). As we mentioned earlier, thus due to these time dependent phenomena, we believe the present spectra seemed also broadened due to intrinsic quantum-mechanical phenomena, such as photoelectron-speed and dynamic polarization dependences of the spectra. We need theoretical calculation of the ARUPS spectra with consideration of time dependent phenomena for discussing the observed results more quantitatively as one of the reviewers commented, but such a theoretical work must be up to theoretical group as the study of the time-dependent photoemission theory which is also an important target in the theoretical field.

The following sentence was added to the main text:

Page 4, line 86 : "The photoelectron spectroscopy monitors the one-hole final state, which is described by a one-hole bound state and a photoelectron in continuum state, hence the term of "hole-phonon coupling" is used in the present manuscript. By temperature-dependent…"

C#5: Minor comments:

- Although generally written clearly, the entire manuscript would benefit from a further round of editing. In particular, there are several grammatical mistakes and missing articles.

- Line 226: "intramolecular" must be "intermolecular"

- Line 440: "300 K" must be "110 K"

- Line 441: "… as a function of sample temperature." should obviously be "… at 110 K.", since

there is no temperature dependent data in Fig. 3b.

- In Fig. 1-3 the colour code must be given.

- Fig. 3c does not share the y-axis with Fig. 3a and b. The y-axis in this case is "intensity" or "normalized intensity" and must be denominated accordingly.

R#5: We corrected the main text according to the reviewer's comment

C#6***: -Line 192 ff: I don't understand the sentence: "At 300 K, a peak at ~15 meV from the top of the HOMO band (H^{Γ}) is visible, which corresponds to the energy of the coupled intermolecular vibrations [9]."***

R#6: The text was changed as follows (page 9 line 197)

"According to the general theory of photoemission spectral function in [9, 18] the position of the peak in the real part (RealΣ) of the quasiparticle spectral function corresponds to the energy of the (*inter*molecular) phonon involved in the hole-vibration coupling [9,18]"

C#7: Line 133ff: It is not clear, how the "Lorentzian component" plotted in Fig. 2c was derived from the fit to a Voigt functions. I understand that the widths of the Lorentzian and Gaussian can be separated, the "Lorentzian component" used by the author seems to be something like and area or spectroscopic weight of the Lorentzian. How should this be derived from the Voigt function, which is a convolution of a normalized Lorentzian and Gaussian?

R#7: The following part was added to the Methods section (page13, line 305 of the revised version)

"In obtaining the plot of Figure 2c, for each $k_{//}$ a Lorentzian curve centred at binding energy x_C, with width w_L and initially arbitrary intensity at peak maximum was plotted. The intensity at peak maximum were adjusted to results in the same integrated area for each Lorentzian, the integral being numerically evaluated in the [x_C-5·w_L; x_C+5·w_L] energy range. For the gap region where two Lorentzian curves were extracted the area were evaluated superimposition of the two curves was considered. The integral were calculated in the (x^{Up}_C-5·w^{Up}_L ; x^{Lo}_C+5·w^{Lo}_L) binding energy range where x^{Up}_C (x^{Up}_L) and w^{Up}_L (w^{Up}_L) are the peak centre (Lorentzian width) of H^U and H^L band components (see Figure 2b)."

Reply to Reviewer #2.

Comment#1: The current research in this topic is well framed in the introduction and the motivation is strong: understanding experimentally the coupling of intra- and inter-molecular motions with the hole. The experiments are highly non-trivial.

Reply#1: We thank the referee for appreciating our experimental efforts in providing high quality ARUPS data of organic single crystal.

C#2: Before giving some detailed comment maybe it is useful for the authors to say that I have the impression that the results are fairly inconclusive possibly because the resolution of the data and the lack of directly comparable theory. I think there is a tendency to find what one wants to find, i.e. signature of coupling with high frequency modes and low frequency modes. However I find neither of them convincing. It is far from obvious that there is a gap in figure 1a (it could be artefact of the intensity diminishing).

R#2: The decrease of the spectral intensity in a well-defined region of the energy/momentum space is a consequence of the gap opening. EDCs broadening due to the i) experimental resolution ii) hole lifetime and ii) structural disorder results in a not zero spectral intensity in the gap region. Moreover the existence of the gap was confirmed by the fitting analysis of the EDCs and variance of the electron momentum, as stated in the main text of the manuscript.

C#3: If there is, its amplitude can be anything between 40 and 200 meV with this resolution. Its origin remains unclear. For once, it doesn't look like the one predicted in ref. [11]. Secondly the high frequency electron-phonon coupling for rubrene are known [Adv. Mater. 17, 1072–1074 (2005)]. These couplings are not too strong and there is a range of modes that contribute in the range 100-200 meV. I find it hard to imagine these result in a gap in the measured ARUP explainable with a single mode. Certainly this deserves a better explanation.

R#3: We would like to recall what explicitly stated in the main text of the paper i.e. "The impact of the molecular vibrations on the electronic properties of organic materials was experimentally investigated by ARUPS for thin films of weakly interacting molecules [20] where non-dispersive HOMO bands consisting of several components with comparable energy separations were observed [20]. Under simplified assumptions (*single mode analysis*),

this energy separation (Ω_0, typically ≳100 meV) **corresponds to the average *intra*molecular vibration (*local* hole-phonon coupling effect) energy while the relative intensities of the HOMO components reflects the coupling strength** [20].”

As stated above the authors are well aware of the limit of the single mode analysis which was however quite successfully applied to describe the UPS-HOMO lineshape a number of organic thin films. Said this, the energy gap value (~140 meV) was extracted by fitting procedure of the ARPES data of rubrene single crystal, in order to reduce the ambiguity in the gap estimation. The as-obtained value was found to be consistent with that obtained *by single mode analysis* of the UPS-HOMO line shape of rubrene gas molecule (~130 meV). In this context the vibrational fine structure of the HOMO lineshape of rubrene gas molecule (i.e. valley-to-peak intensity modulation) is reflected into gap opening (i.e. intensity modulation) in the single crystal HOMO band dispersion as predicted in Ref.[11] for 1D pentacene system. The obvious difference between results of ref.[11] and the present results are to be ascribed to the difference in molecules (pentacene vs. rubrene), system structure and dimensionality (1D vs 3D). A detailed calculation of quasiparticle spectral function of the rubrene singe crystal with inclusions for example of additional mode would surely be beneficial to improve the agreement with experimental data, but are beyond the scope of the present paper. In this context our experimental results can surely stimulate the interest of theoretical community in the field.

C#4: An analogy with ref. [9] is used to explain the possible role of intramolecular modes. The material is so different that this analogy is not clear at all.

R#4. Ref. 9 is cited to generally illustrate the impact of phonons on the electronic band dispersion of a many electron system as measured by ARPES. i.e. the coupling between vibrations and quasiparticle states originating upon photoemission can results in a kink structure in the measured band dispersion. The fact that ref. 9 is referred to metal case is simply because similar effects were not yet observed in organic materials as mainly due to the limited ARPES energy/momentum resolution and limited sample quality. In this context the present results represent the first successful measurements of this kind for organic single crystal.

C#5: It was part of the motivation of this work to investigate a system where traditional band transport concepts fail but the same ideas are stretched to explain the results in terms of increased effective mass. It would be much better to present a model that reproduce directly the experimental results rather than using the model to extract information that combine **experimental data and assumptions of the model.**

R#5: The main goal of this work was to investigate the electronic properties of high quality organic single crystal with unprecedented energy and angular resolution with the aim to clarify the impact of molecular vibrations on the band dispersion. Despite theoretical predictions and their relevance for transport properties, in fact, charge-phonon related effects in organic single crystal were yet to be directly observed.

In this paper we demonstrate that (i) charge-phonon coupling effects in the organic single crystal exist and (ii) they can lead to significant modification of the band dispersion with respect to DFT based calculation where these effects are normally not included. This difference are highlighted in an increase of the hole effective mass (m*) with respect to theoretical estimation, where m* can be seen as a parameter to quantitatively describe the effect of intermolecular phonons on the charge localization. The theoretical studies of this issue are still in progress, and developing a charge transport model was obviously beyond the scope of this work. The main aim was to provide a solid experimental validation of the impact of the phonons on the charge localization in organic single crystals, which can help and supports necessary theoretical investigations

C#6: The raw results do not show clear temperature dependent effect and the data presented in Figure 4 are not very clear to me.

R#6: We have given additional ARUPS data in the supplemental materials to highlight the temperature dependence (see Figure S3 of SM). About Figure 4 issue, its content is already extensively described in the text and in the corresponding caption. For clarity purpose, the details of the parabolic fitting procedure for effective mass extraction were better described as follows (Page 14, line 328 of the revised text)

"A value of $1.4m_0<m^*<2.4m_0$ was found, considering the binding energy and wave vector uncertain in the HOMO peak positions. Representative parabolic fitting curves for the m* upper and lower limit are reported in Fig. 2b. The same fitting method was applied to the theoretical band dispersion [16] within the same wave vector range. A value of $m^*=0.9m_0$

was extracted, which is consistent with the theoretical estimation in ref. 16 ($0.9m_0$) and in good agreement with previous reports ($\sim0.8m_0$) [10,15].”

Reply to Reviewer #3

Comment#1: Measurements of the HOMO band dispersion of rubrene single crystals have been quite often reported in the literature (several by some of these authors). The data here is however of very high quality and resolution allowing hitherto unseen fine structure to become apparent. These bear a strong resemblance to phenomena proposed, from theoretical deliberations in ref 11, to arise from hole-phonon coupling and the data was analysed accordingly. The regions critical to their interpretation are the gap at ~0.22 A^{-1} and a kink at around 0.065A^{-1} assigned to the effects of intra- and intermolecular vibrations, repectively. Because of it significance to charge transport in organics I believe the work warrants publication if the following points are considered.

Reply#1: We thank the referee for appreciating our experimental efforts in providing high quality ARUPS data of organic single crystal.

C#2: What I find puzzling is that two bands are expected but "no experimental evidence" for the 2nd band (H2) was found. Vollmer et.al (J.El.Spec.185(2012)55) do seem to see both bands with the expected separation near gamma of ~0.25 eV. Without an understanding of why the H2 is missing the gap opening argument is weakened. For instance, it might be argued that in the first half of the Brillouin zone the first band is strong while in the 2nd half the H2 dominates – then the observation would not be gap opening in a single band.

R#2: This comment is basically similar to the other reviewer. We have answered to this comment, but again summarise the reason below. The identification of upper H^U and lower H^L experimental band with the H_1 and H_2 band, respectively, suggest a possible modulation of the H_1, H_2 band intensity, as possibly related to some kind of photoemission matrix effect. However, in the present case this effect can be excluded on the basis of the following arguments:

(a) In ARUPS investigation of single crystal materials, rapid spectral intensity changes at variance of wavevectors component are generally observed around the high symmetry points of the Surface Brillouin Zone (SBZ). This intensity modulation can be ascribed to photoemission matrix element effect depending in turn on (i) the wavefuntions symmetry, (ii) incident light polarization (iii) the photon energy and (iv)

the experimental geometry. In the present case of rubrene single crystal, the suggested intensity modulation of the H_1 and H_2 band would occur within the SBZ where no high symmetry point, by definition, exists. This makes the proposed identification of the H^L band with H_2 band quite unlikely.

In principle, additional high symmetry points can be introduced by in plane crystal reconstruction in the bulk and/or surface region. However change in the in-plane lattice periodicity of the bulk region is not supported by any reported X-ray diffraction data. Moreover, high-resolution frequency modulation atomic force microscopy investigation of rubrene single crystal excluded the occurrence of any surface reconstructions [Applied Physics Letters 95, 093302 (2009)].

(b) The band structure calculations predict the existence of two dispersive bands along the ΓY direction of rubrene single crystal. This is confirmed by previous ARUPS reports on rubrene single crystal [Phys. Rev. Lett 104, 156401 (2010), Appl. Phys. Express 5, 111601 (2012)]. At Γ point the experimental energy separation between the H_1 and H_2 band is ~0.30 eV [Appl. Phys. Express 5, 111601 (2012)] with respect to a theoretical value of ~0.2 eV. In this context, while the theoretical calculations reproduce quite well the H_1 experimental band dispersion, the bandwidth of the H_2 band is slightly overestimated. In order to reproduce the energy separation at Γ point the H_2 theoretical band dispersion must be ~30% compressed along the binding energy axis. This result is a further deviation of the H_2 band with respect to the experimentally measured “lower” (H^L) sub-band of rubrene single crystal. In this context the identification of the H^L with H_2 band as modulated by cross section effects (if any) is unlikely.

(c) It is worth to note in passing that the larger energy separation between observed HOMO derived sub-bands, a and b, with respect to the theoretical predictions (see refs. 16 and 17) were discussed by Ciuchi et al. (see ref. S4 in SM) for pentacene single crystal case as a consequence of the hole coupling with the intramolecular vibrations.

The Supplemental Materials were modified accordingly to include the points of the above discussion. Moreover we would like to comment in the following on the absence of any visible band H_2 in our experimental data.

In organic systems with two inequivalent molecules per unit cell, the HOMO band is split into two sub-bands and the UPS peak intensity and width of the two HOMO-derived bands is generally different [See Ref. 4 in the main text]. These differences can be tentatively ascribed to matrix element effect in the photoemission process as related to the symmetry of the responsible wavefunctions, energy and polarization of incoming photons and emission direction of the photoelectrons [See Ref. 4 in the main text]. In case of single or weakly interacting molecules the angular distribution of the photoelectron intensity at variance of the photon energy is well theoretically understood [Nat. Comm. **5**, 4156 (2014)] and it is closely related to the symmetry of the molecular orbitals in the nearly isolated molecules. For organic solids the situation is more complex as mainly due to the spatial delocalization of the HOMO wavefunctions and a complete understanding of the photoelectron angular distribution of UPS intensity has yet still to be provided.

In previous ARUPS investigation of rubrene single crystal, two HOMO derived subbands (H_1, H_2) were reported as resulting from the presence of two inequivalent molecules per unit cell [Phys. Rev. Lett 104, 156401 (2010), Appl. Phys. Express 5, 111601 (2012)]. **The observed UPS measured intensity of the higher binding energy band (H_2) is generally lower (<40%) than that of the H_1 band and it is clearly visible mainly in the nearby of the Γ point.** These differences were observed in a wide range of photon energy (20~40 eV) as well in correspondence of various UPS experimental geometry. As mentioned above the differences can be ascribed to photoemission matrix effect reflecting the different spatial distribution of the H_1 and H_2 wavefunctions. A detailed understanding of this intensity difference is beyond the current theoretical understanding of the photoemission process in organic solids and certainly beyond the scope of the present contribution.

C#3: The disappearance of the kink in going to low temperatures is critical to this work however it is not compellingly presented in the data. EDCs at 300 and 110K in the region of the kink (~0.065A-1) need to be added in Fig3c so that the reader may judge whether the kink has indeed disappeared at 110K.

R#3: A set of EDCs at various $k_{//}$ were added in the SM (section 2) to show the binding energy shift of EDCs' peak as induced by temperature reduction. The following sentence was added to the text (Page 7, line 149 of the revised manuscript):

“A comparison between the EDCs as obtained at 300K and 110K in the [0~0.095] $Å^{-1}$ wavevector range is shown in Figure S3 of SM to illustrate the temperature induced binding energy shift resulting in the quenching of the kink structure.”

C#4: Minor points:

C#4(a): The colour chosen for the data points in the inset of fig. 3b make them almost invisible. The theoretical bands should be smooth and not kinked as they appear in the figures.

R#4(a): The data points and plot were modified accordingly.

C#4(b): The schematic of the real space unit cell in Fig 1a is too small to be useful – it should be also added to supplementary information with a reasonable size.

R#4(b): We believe that the size of the figure is suitable for reader unless different advice during editorial revisions.

C#4(c): Fig 1(d) is almost redundant- if they have the data it should be extended to include the BZ boundary (Y).

R#4(c): The data are useful for qualitative and introductory discussion of the rubrene band dispersion. We prefer to maintain as it is.

A full dispersion was measured for another crystal as in SM (SC#3). This is simply to confirm the overall band dispersion behaviour as reported in previous investigations on rubrene single crystal by some of the authors of the present contribution (Ref.22 of the main text).

REPLY to REVIEWERS' COMMENTS

Dear Editor,

We thank the reviewer for the useful comments and their help in improving the quality of the manuscript. In the following a detailed reply to their comment is included

With Best Regards

Fabio Bussolotti

(on behalf of the author)

-Reply to Reviewer #1 (Remarks to the Author):

Comment. ***In the revised version of their mansucript the authors have taken all critics raised in my original report into account. They have taken care to correct all minor mistakes and thoroughly replied to all comments. I consequently support publication of this manuscript in Nature Communications without further revision.***

We thank the referee for having appreciated the quality of our work.

Reply to Reviewer #2 (Remarks to the Author):

Comment. ***In essence this paper reports top quality experimental results with some plausible but, to some extent, non-quantitative explanation of the observations. On balance I think this work should be published on Nature Communications possibly with the inclusion in the main manuscript of some of the comments made by the authors in the response to the referees about the lack of quantitative theory to explain these experiments.***

Essentially two new features are observed one of which temperature dependent in the 100-300 K range. It would be nice if the temperature in-dependent effect is due to high frequency phonons and the temperature dependent one is due to low frequency phonons, and this is their main point. The latter is certanly true, and the former it possibly true.

The observation itself does not allow labelling the hole-phonon coupling as local or non-local, this derives from calculations showing the the non-local coupling is important at low frequency and the local coupling is important at high frequency.

Even less based on the observation is the labelling of the low frequency modes effect as "inter-molecular" mode. The modes can be very well intramolecular and affect the transfer integral, i.e. be non-local.

My recomendation is therefore to publish this work on NC with few additional sentences highlighting what are the most speculative aspects of this paper and those requiring more quantitiative theoretical work. It should become clear that this paper represents an important step forward rather than achieveing a complete understanding of the hole-phonon coupling effect.

We thank the referee for appreciate our experimental efforts in providing high quality experimental data. We are well aware of the limit of present attribution of the observed modification of the HOMO band as induced by molecular vibrations which is consistently based, nonetheless, of the present state of the art of theoretical research in the field. In this context the importance (and the limit) of our paper as solid experimental basis for further theoretical investigation in the field are stated in the conclusive part which are rewritten as follows to partly recall the concept already included in previous reply:

"In conclusion, in this paper we report on the electronic properties of rubrene single crystal as investigated by ARUPS and demonstrate, for the first time, the impact of the molecular vibrations on the band dispersion of an organic molecular semiconductor. Evidences of the hole coupling with both the intramolecular and intermolecular phonons manifest in local change of the HOMO band curvature in the energy/momentum space. These findings were discussed in terms of their impact on the hole dynamics in the rubrene single crystal and they were found to depend on the energy of vibrational modes with respect to the hole transport level position i.e. top of the HOMO band. Hole coupling with the intramolecular phonons (~140 meV) is expected to have no significant impact of the transport properties, while the interaction with intermolecular vibration (~10 meV) leads to hole localization. This result suggests the main role of non-local hole-phonon coupling effects in determining the charge transport mechanism and the temperature dependence of the hole mobility in rubrene single crystal. As the energy of vibrational modes for various organic materials lies in a comparable energy range (~10-200 meV) [4] the above findings can be generalized to the wide class of organic molecular semiconductor to enlighten the nature of the charge transport mechanism in this technological important class of materials."

was changed as:

"In the present research we report a rigorous experimental investigation on the electronic properties of rubrene single crystal, a prototypical example of organic semiconductor. We

provided a clear evidence of peculiar features in the band dispersion which are not predicted by usual theoretical band structure calculations. In analogy with ARUPS theory, this deviation strongly resembles the effect of charge-phonon coupling in one-hole state as commonly reported in ARUPS investigation of inorganic single crystal. The temperature dependent data supported this interpretation. Moreover this is also consistent with the current state of the art of quasiparticle spectral function calculation in organic systems, with obvious deviations as related to the difference between our investigated system and the simplified assumption in the theoretical model. We also provide direct evidence on how charge-phonon coupling may increase charge localization, well above the limit predicted by DFT band structure calculation, which is relevant results for applications. In this context a complete simulation of the quasiparticle spectral function with theoretical calculation of the UPS spectra as well as a more quantitative modelling of charge transport in organic materials will help in a full rationalization of the present experimental results. In this context, the present work can attract interest of theoretical groups and stimulate the theoretical debate thus representing an important step towards the full understanding of the impacts of charge-phonon coupling in organic semiconductor crystals

----

Reviewer #3 (Remarks to the Author):

Comment. ***The responses are just satisfactory. The addition of Figure S3 was necessary- I guess some readers will believe the kink removal others not - I tend to be a believer. Interpretations can change whats important is that the data stands the test of time- and I feel the community will not get better data to work with for a number of years. All referees were disturbed by the missing H2 band - the answer of matrix effects is of course true but says nothing- the reason I wanted to see the real space orientation was so I could think about reasons for H2s vanishing intensity (and for that one needs to know the experimental geometry, including the molecules orientations). Your Response "we believe that the size of the figure is suitable for reader" was therefore extremely irritating and clearly wrong as I am a reader!***

We thank the referee for the help in improving the paper quality and we apologize for not consider his/her suggestion in a proper way. The Figure 1 was modified as follows:
The schematic sketch of the rubrene molecule was moved as inset in the original panel (d). The panel (b) was enlarged to improve the visibility and the different parts renamed as (a) and (b). The other figure panel were renamed accordingly in the caption and through the whole manuscript.
